# Rapid deployment technology versus conventional sutured bioprostheses in aortic valve replacement

**DOI:** 10.1111/jocs.16223

**Published:** 2022-01-14

**Authors:** Mohammad Yousuf Salmasi, Sruthi Ramaraju, Iqraa Haq, Ryan A. B. Mohamed, Taimoor Khan, Faruk Oezalp, George Asimakopoulos, Shahzad G. Raja

**Affiliations:** ^1^ Department of Surgery Imperial College London UK; ^2^ Department of Cardiac Surgery Royal Brompton and Harefield Trust London UK

**Keywords:** aortic valve replacement, rapid deployment valves, sutureless valves

## Abstract

**Objectives:**

Despite the benefits of rapid deployment aortic valve prostheses (RDAVR), conventional sutured valves (cAVR) are more commonly used in the treatment for aortic stenosis. Given the paucity of randomized studies, this study aimed to synthesize available data to compare both treatment options.

**Methods:**

A systematic search of Pubmed, OVID, and MEDLINE was conducted to retrieve comparative studies for RDAVR versus cAVR in the treatment of aortic stenosis. Out of 1773 returned titles, 35 papers were used in the final analysis, including 1 randomized study, 1 registry study, 6 propensity‐matched studies, and 28 observational studies, incorporating a total of 10,381 participants (RDAVR *n* = 3686; cAVR *n* = 6310).

**Results:**

Random‐effects meta‐analysis found no difference between the two treatment groups in terms of operative mortality, stroke, or bleeding (*p* > .05). The RDAVR group had reduced cardiopulmonary bypass (standardized mean difference [SMD]: −1.28, 95% confidence interval [CI]: [−1.35, −1.20], *p* < .001) and cross‐clamp times (SMD: −1.05, 95% CI: [−1.12, −0.98], *p* < .001). Length of stay in the intensive care unit was also shorter in the RDAVR group (SMD: −0.385, 95% CI: [−0.679, −0.092], *p* = .010). The risk of pacemaker insertion was higher for RDAVR (odds ratio [OR]: 2.41, 95% CI: [1.92, 3.01], *p* < .001) as was the risk of paravalvular leak (PVL) at midterm follow‐up (OR: 2.52, 95% CI: [1.32, 4.79], *p* = .005). Effective orifice area and transvalvular gradient were more favorable in RDAVR patients (*p* > .05).

**Conclusions:**

Despite the benefits of RDAVR in terms of reduced operative time and enhanced recovery, the risk of pacemaker insertion and midterm PVL remains a significant cause for concern.

## 1 INTRODUCTION

Surgical aortic valve replacement (AVR) remains the established treatment modality for severe aortic valve stenosis. The introduction of rapid deployment AVR prostheses (RDAVR), particularly sutureless valves, in the last two decades has offered an alternative technique implantation during surgical AVR. A panel of international experts on sutureless, rapid deployment and stented bioprostheses recommended its use for isolated AVR in elderly patients with severe comorbidities and aortic wall conditions such as calcified root and porcelain aorta.^1^ Other benefits of RDAVR in comparison to conventional sutured AVR (cAVR) include reduced operation time with reduced cross‐clamp and cardiopulmonary bypass times, as well as favorable effective orifice area (EOA) and hemodynamic outcomes.^2^, ^3^

Current guidelines do not make specific recommendations for the use of RDAVR.^4^, ^5^ The 2020 American Heart Association (AHA) and American College of Cardiology (ACC) and the 2017 European Society of Cardiology (ESC) and the European Association for Cardio‐Thoracic Surgery (EACTS) guidelines maintain that cAVR is the gold standard for the replacement of aortic valves in severe aortic stenosis for patients under the age of 65 (AHA/ACC) and under the age of 75 (ESC/EACTS). With the emerging evidence of the use of transcatheter aortic valve implantation (TAVI), both guidelines are increasingly recognizing its use as a noninferior alternative for high‐risk patients and patients over the age of 65 with TAVI as a preferred option in some cases.^4^

There have been only two randomized controlled trials (RCTs) comparing RDAVR with conventional AVR, Borger^6^ (*n* = 46 RDAVR [Intuity] vs. 48 cAVR) and Dedeilias^7^ (*n* = 25 RDAVR [Perceval] vs. 25 cAVR). Operative times were reported to be significantly lower in the RDAVR groups in both studies. Only the study by Borger and colleagues reported on paravalvular leak (PVL) and found there to be no significant difference between the two groups.^8^

In the current era of evidence‐based medicine, pairwise meta‐analyses are increasingly used to synthesize the results of different trials evaluating the same intervention(s) to obtain an overall estimate of the treatment effect of one intervention relative to the control.^9^ Given the paucity of comparative data between the two bioprosthetic classes for the surgical management of aortic stenosis, data synthesis of available evidence will help provide a more robust analysis. This systematic review and meta‐analysis aims to comprehensively compare postoperative outcomes in patients undergoing cAVR and RDAVR.

## 2 METHODS

This study was conducted in accordance with the Prescribed Reported Items for Systematic Reviews and Meta‐analysis (PRISMA)^10^ and Assessing the methodological quality of systematic reviews (AMSTAR) Guidelines. Ethical approval was not required to conduct this study, neither was informed consent.

### 2.1 Search strategy

The EMBASE AND MEDLINE databases were searched for relevant articles published after January 1, 2000 were, thus taking into account the implementation of RDAVR prostheses. Search terms incorporated “aortic valve replacement” or derivatives of its terms (e.g., prosthesis, surgery, and AVR). “Rapid‐deployment” or “sutureless” were terms added to the search, as well as specifically named prostheses (Perceval, Intuity, 3F Enable). The full search strategy can be found in the Appendix A (I).

### 2.2 Screening

Selected articles were screened for title and abstract by two reviewers (S. R. and I. H.), and conflicts were resolved through discussion. Selected studies were checked by a third reviewer (M. Y. S.). Studies were included from the full‐text screening if there was a full‐text article comparing cAVR and sAVR at least for the intraoperative parameters (e.g., cardio‐pulmonary bypass time, cross‐clamp time, etc.), and patient demographics for each group. Both retrospective and prospective studies were considered. Case reports were excluded.

### 2.3 Inclusion and exclusion criteria

#### 2.3.1 Inclusion

Only studies in the English language were considered, and studies on patients above 18 years of age at the time of the operation. Studies incorporating two comparable treatment groups, receiving RDAVR in one study arm and cAVR in the other, were considered. Conventional AVR protheses were selected based upon the mention of any of the leading market prostheses (e.g. perimount, Trifecta, etc). For RDAVR, studies incorporating either (or a combination) of the known prostheses by name (perceval, intuity, 3F‐enable) were included. Studies reporting measurable short and/or long‐term outcome data were considered.

#### 2.3.2 Exclusion

Case reports were excluded. Studies that lacked clarity in design or where separation between covariates/outcomes of the RDAVR and cAVR groups were not considered. Papers displaying an element of bias thus favoring one group over the other were excluded. Study cohorts that consisted of any non‐AVR patients were not strictly excluded, unless non‐AVR patients were included along with AVR patients and were not analyzed as separate groups.

### 2.4 Outcome measures

These were divided into three categories:

*Operative measures*: cardiopulmonary bypass time, aortic cross‐clamp time.

*Short‐term outcomes*: mortality, stroke, bleeding, pacemaker implantation, hospital/intensive treatment unit (ITU) stay.

*Echocardiographic outcomes*: PVL, indexed effective orifice area (iEOA), transvalvular gradient (peak and mean).

### 2.5 Data extraction

Data from selected studies following full‐text screening were extracted according to a structured protocol into predefined a summary table, which included the headings for study characteristics, patient characteristics, and preoperative, intraoperative and postoperative data. The full table and all the headings can be found in the Appendix A.

### 2.6 Statistical analysis

The Newcastle–Ottawa Scale was used to assess the quality of nonrandomized studies, with particular focus on comparability of patient groups and patient selection bias. It is a well‐validated and standardized screening tool for the risk of bias.

The odds ratio (OR) was used as the summary statistic for binary outcomes (e.g., mortality, stroke, and pacemaker insertion) whereas continuous outcomes (e.g., hospital stay and transvalvular gradient) were analyzed using reported means and standard deviations (SD) thus yielding a standardized mean difference (SMD). A random‐effects model was chosen over a fixed‐effects model due to the expected heterogeneity between the studies. Heterogeneity was investigated using Cochrane's test and the *I* ^2^ statistic. Funnel plots were generated to assess for publication bias. Peter's test for small studies was conducted to rule out large effects from potentially nonsignificant studies. Meta‐regression analysis was used to investigate the effects of covariates, including patient and operative characteristics. Statistical analyses were conducted using the Stata 13.0 software (Stata Corp.).

### 2.7 Subgroup analysis

As well as pooled analysis of all studies, meta‐analyses for all outcome measures were repeated in subgroups of studies based upon factors of design or prostheses used. This helped account for any perceived study heterogeneity. Subgroup categories included as follows:

1. Study design: (retrospective, propensity matched, RCT, registry).
2. RDAVR prosthesis (Perceval, Intuity, 3F Enable, Mixture).
3. cAVR prosthesis (Perimount, non‐Perimount, Mixture).
4. Surgical approach (Sternotomy, mini‐sternotomy, thoracotomy, Mixture).

## 3 RESULTS

**Figure 1:**
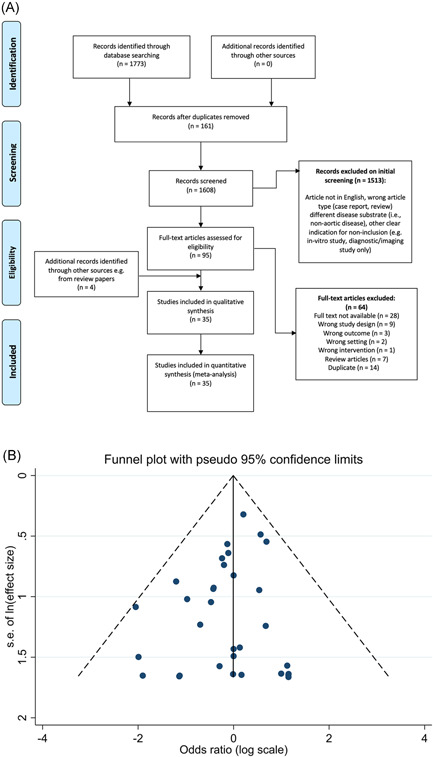
(A) PRISMA flowchart of included studies at each stage of screening. (B) Funnel plot for included studies. PRISMA, Prescribed Reported Items for Systematic Reviews and Meta‐analysis

From our search, we identified 1608 articles, of which 1513 were excluded based on title and abstract screening, as shown in Figure 1A. Full texts were obtained for the remaining 95 articles, and 35 were included in our final analysis, satisfying the inclusion/exclusion criteria. Study characteristics of the included papers are shown in Table 1.

**Table 1:** Characteristics of included studies

Author	Year	Study design	Total no. of participants	No. RDAVR	No. cAVR	Mean follow‐up (years)	Follow‐up, years (RDAVR)	Follow‐up, years (cAVR)	Mean age (RDAVR)	Mean age (cAVR)
Andreas	2016	Parallel cohort	248	116	132		2	2.9	75	70
Beckmann	2016	Comparative, retrospective	128	92	36		2.9 ± 1.6	4.4 ± 2.8	62	79
Belluschi	2017	Observational, retrospective	124	62	62				79	79
Bening	2017	Retrospective	68	43	25				74.1	74.2
Casha	2018	Retrospective, single‐center, risk‐matched cohort.	40	20	20				76.95	78.29
Chiariello	2019	Retrospective, nonrandomized trial	76	52	24	2.9 ± 0.5			78.5	77
D'Onofrio	2013	Propensity‐ matched analysis	286	31	112		0.083		73.5	73.5
Dalen	2015	Observational, retrospective	565	182	383	3.2 ± 2.1			77.5	73.8
Dedeilias	2016	Prospective, randomized	50	25	25		0.67 ± 0.13	0.67 ± 0.15	80	79
Ensminger	2018	Observational, propensity‐matched, prospective study, multicentre study (~22,000 patients)	2042	1021	1021				75	75
Ferrari	2017	Retrospective, non‐randomized trial	64	32	32	1	1	1	78	72.5
Forcillo	2016	Retrospective	395	76	319				83	83
Ghoneim	2016	Retrospective, single‐center study	351	49	259				78	74.7
Gilmanov	2014	Retrospective, observational, cohort	266	133	133		1.28 ± 0.67	4.47 ± 2.42	75.3	73.6
Gotzmann	2019	Retrospective, propensity score matched single‐center study	108	54	54	0.77			73.2	72.9
Hanedan	2018	Retrospective, non‐randomized study	70	38	32	2.19 ± 1.76	1.41 ± 0.97	3.12 ± 2.03	71.2	69.5
Ilhan	2020	Prospective, cohort study	140	48	92		0.5	0.5	76.3	73.6
Konertz	2017	Retrospective, cohort study	79	16	63				73	67.5
Mujtaba	2018	Retrospective, observational, cohort	763	139	624				74.3	71.74
Muneretto	2015	Retrospective, propensity matched, cohort	408	204	204	2	2	2	80	79
Muneretto	2014	Prospective, cohort study	108	53	55	2	2	2	79	79
Nguyen	2017	Retrospective, observational	236	59	177	1	1	1	70	69
Pollari	2014	Retrospective	164	82	82	1.08 ± 0.5			75.5	74.5
Rahmanian	2018	Nonrandomized, retrospective analysis	326	163	163				75.8	75.8
Sainte	2017	Retrospective, single‐center, matched case‐control	104	52	52	1			79.1	78.5
Santarpino	2013	Multicenter study	100	50	50				77.5	71.7
Shalabi	2016	Prospective, cohort study	44	22	22		1.17 ± 0.92	3.75 ± 1.83	77	79
Shreshta	2013	Retrospective, multicentre, nonrandomized	120	50	70	2.73 ± 1.29				79.8
Smith	2017	Retrospective, propensity‐matched cohort study	82	41	41	0.14 ± 0.11				76.5
Stanger	2017	Retrospective, observational	1388	82	1306				76.9	72.6
Stegmeier (Labcor)	2020	Retrospective	87	25	62				79	74.4
Thitivaraporn	2018	Retrospective	20	10	10	1			81.5	81.1
Villa	2019	Retrospective	231	113	118				80.1	75.5
Vola	2015	Retrospective, single‐center	83	41	42	2.13 ± 1.08	1.59 ± 0.42	2.59 ± 1.12	75.7	75.3
Wahlers	2018	Retrospective, multicentre, nonrandomized, propensity‐matched cohort	545	287	258	3	2.7 ± 0.8	3	75.3	68.5

Abbreviations: cAVR, conventional aortic valve replacement; RDAVR, rapid deployment aortic valve replacement.

Of the 35 included studies, 21 studies were perceval only,^7^, ^11^, ^12^, ^13^, ^14^, ^15^, ^16^, ^17^, ^18^, ^19^, ^20^, ^21^, ^22^, ^23^, ^24^, ^25^, ^26^, ^27^, ^28^, ^29^, ^30^ 6 studies were INTUITY only,^31^, ^32^, ^33^, ^34^, ^35^, ^36^ and the rest had a mixture of RDAVR valves.^37^, ^38^, ^39^, ^40^, ^41^, ^42^, ^43^, ^44^ Most (28) of the included studies were retrospective,^11^, ^12^, ^13^, ^14^, ^15^, ^16^, ^17^, ^18^, ^19^, ^20^, ^21^, ^23^, ^24^, ^26^, ^28^, ^29^, ^30^, ^32^, ^33^, ^34^, ^35^, ^36^, ^37^, ^39^, ^40^, ^42^, ^43^, ^44^ and the rest^7^, ^22^, ^25^, ^27^, ^31^, ^38^, ^41^ were prospective. Eight of the studies were propensity‐matched.^13^, ^21^, ^26^, ^30^, ^35^, ^38^, ^39^, ^43^ Only 1 study was randomized.^7^ More detailed information on study characteristics are shown in Table 1 and the Appendix A.

### 3.1 Publication bias

Publication bias was assessed using the Peter's test. The results of the analysis are shown in the funnel plot in Figure 1B. There was no evidence of publication bias and all the studies fall within the pseudo‐95% confidence interval. The effect of small studies was nonsignificant (*p* = .115).

The inherent risk of bias in the studies was assessed using a modified version of the Newcastle–Ottawa Scale. It assessed the quality of studies based on the selection of patients for either of the two treatment groups, the quality of the description of the surgical procedures performed and the comparability of the two cohorts or lack thereof (Appendix A). Although the studies varied in quality, all included articles scored well on the scale.

### 3.2 Operative time

#### 3.2.1 Cross‐clamp time

Twenty‐one studies^20^, ^21^, ^22^, ^23^, ^25^, ^26^, ^27^, ^28^, ^29^, ^30^, ^32^, ^35^, ^36^, ^39^, ^40^, ^41^, ^42^, ^43^, ^44^ of the included studies found a shorter aortic cross clamp time (AXT) in the RDAVR group compared with cAVR, as reflected in the overall pooled analysis (SMD: −1.28, 95% CI: [−1.35, −1.20], *p* < .0001), when stratified by sutureless valve type, by study design and by surgical approach. However, there was considerable heterogeneity (*I* ^2^ = 92.2%), both overall and within subgroups.

#### 3.2.2 CPB time

Twenty‐two studies^7^, ^11^, ^14^, ^16^, ^20^, ^21^, ^22^, ^23^, ^25^, ^26^, ^27^, ^28^, ^29^, ^32^, ^35^, ^36^, ^39^, ^40^, ^41^, ^42^, ^43^, ^44^ included data on cardiopulmonary bypass (CPB) time. Sutureless valves had a shorter CPB time overall (SMD: −1.04, 95% CI: [−1.12, −0.97]), when stratified by sutureless valve type. In addition, perceval, intuity, 3 F enable and studies which used a mixture of valves independently showed shorter CPB times.

### 3.3 Postoperative complications

#### 3.3.1 Mortality

**Figure 2:**
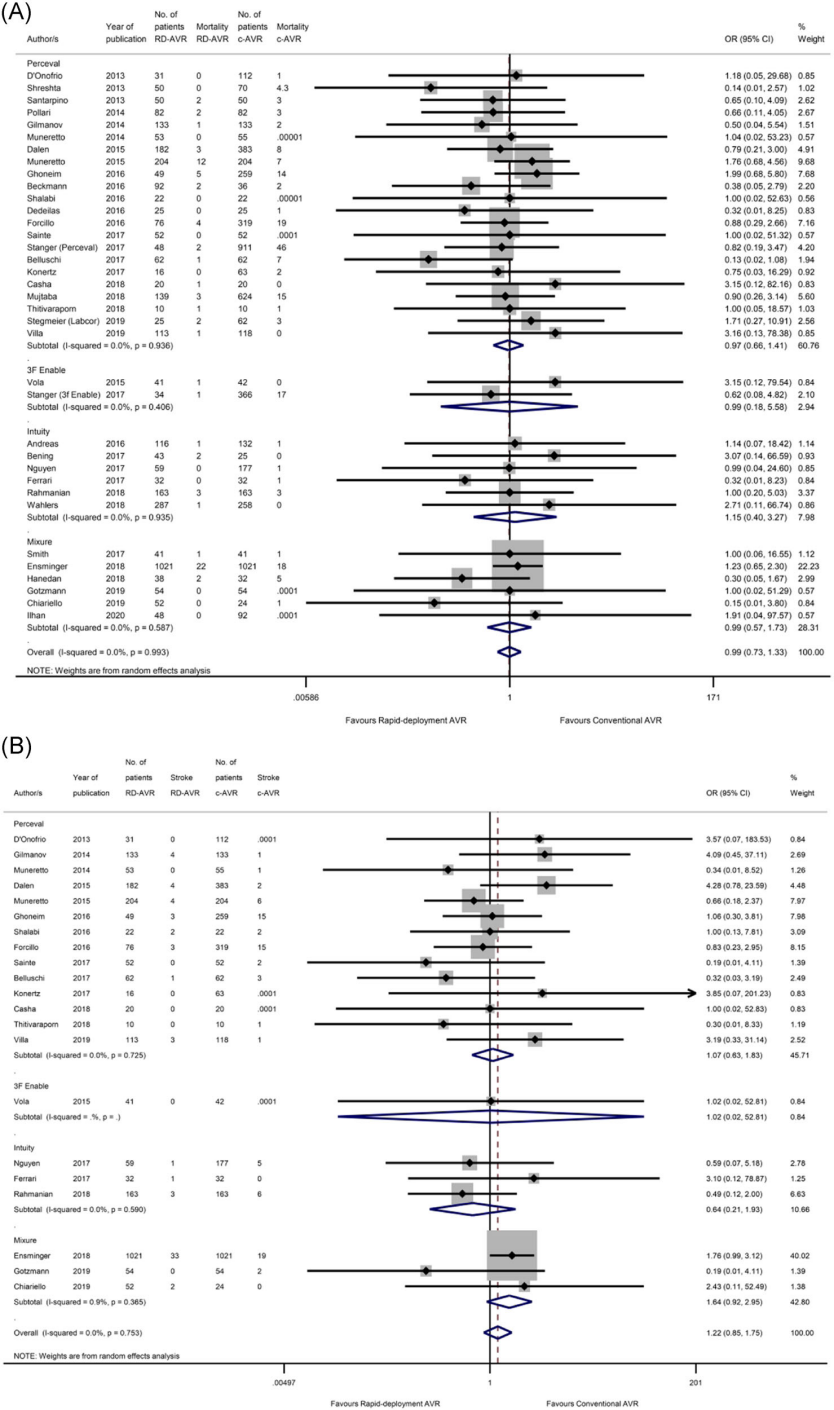
(A) Forest plot demonstrating the operative mortality following RDAVR versus cAVR. Subgroup analysis is shown based on the RDAVR prosthesis used in each study. (B) Forest plot demonstrating the risk of stroke following RDAVR compared with cAVR. Subgroup analysis is shown based on the RDAVR prosthesis used in each study. cAVR, conventional aortic valve replacement; RDAVR, rapid deployment aortic valve replacement

All the included studies reported mortality data. Mortality did not differ between the sutured and sutureless groups when stratified by operation approach, valve type or study design. Overall, there was no significant difference in mortality between the sutureless and conventional groups (odds ratio [OR]: 0.99, 95% confidence interval [CI]: [0.73, 1.33], *p* = .933) (Figure 2A). The median sternotomy approach tended toward favoring RDAVR (OR: 0.821, 95% CI: [0.47, 1.41, *p* = .476]. Intuity valves trended toward favoring cAVR, while Perceval and 3 F enable studies showed little difference between the two groups. Subgroup analysis by dividing the studies into perimount valve insertion, nonperimount valve insertion and mixture showed no difference in mortality, except for the nonperimount group tending toward favoring sutureless (OR: 0.58, 95% CI: [0.23, 1.48], *p* = .253). Overall, the studies were homogenous (*I* ^2^ = 0.0%, *p* = .993), and subgroup analysis also showed consistent homogeneity.

#### 3.3.2 Stroke

Twenty‐one studies reported the incidence of stroke in their cohorts.^12^, ^13^, ^15^, ^16^, ^17^, ^18^, ^21^, ^24^, ^26^, ^27^, ^29^, ^30^, ^32^, ^33^, ^34^, ^37^, ^38^, ^39^, ^44^ Overall incidence of stroke was not significantly different between the two arms (OR: 1.22, 95% CI: [0.85, 1.75], *p* = .277) (Figure 2B). When stratified by sutureless valve type, studies which had a mixture of RDAVR prosthesis types showed a greater difference between cAVR and RDAVR (OR: 1.64, 95% CI: [0.92, 2.95], *p* = .096) compared with studies which investigated just intuity, 3 F enable or perceval in isolation. When subdivided according to study type, the registry study tended toward favoring cAVR (*p* = .052), but this was not supported in other study types. Overall, the studies were homogenous (*I* ^2^ = 0%).

#### 3.3.3 Bleeding

Nineteen studies^14^, ^15^, ^16^, ^17^, ^18^, ^20^, ^21^, ^22^, ^23^, ^24^, ^29^, ^31^, ^32^, ^35^, ^37^, ^38^, ^39^, ^40^, ^43^ included information about bleeding. Bleeding was not significantly different between conventional and rapid deployment AVR, although the pooled studies showed a slight trend toward favoring cAVR (OR: 1.08, 95% CI: [0.74, 1.59], *p* = .687). When stratified by study design, sutureless valve type and surgical approach, only the registry study subcategory showed significance (OR: 0.70, 95% CI: [0.56, 0.89], *p* = .003). However, there was only one study in this category.

### 3.4 Pacemaker implantation

**Figure 3:**
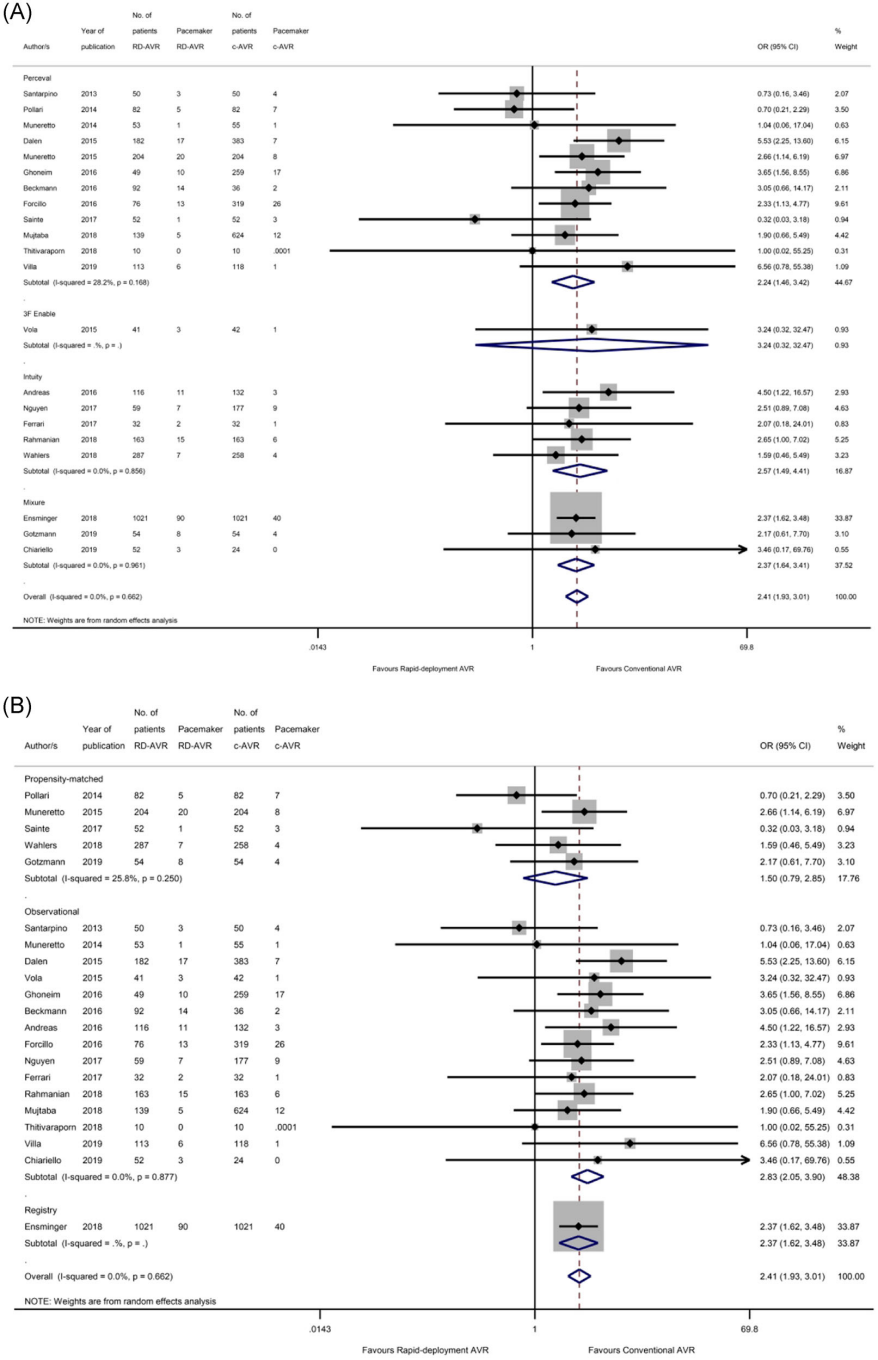
Forest plot demonstrating the risk of pacemaker insertion following RDAVR compared with cAVR. Subgroup analysis is shown based on (A) the RDAVR prosthesis used in each study, and (B) the study design. cAVR, conventional aortic valve replacement; RDAVR, rapid deployment aortic valve replacement

There was a statistically significant difference in pacemaker implantation between the two arms which favored cAVR when conducting a pooled analysis of all studies (OR: 2.41, 95% CI: [1.93, 3.01], *p* < .0001) (Figure 3). Subgroup analysis found this to be consistent with the (12) Perceval‐only studies^11^, ^14^, ^15^, ^16^, ^19^, ^21^, ^22^, ^23^, ^24^, ^25^, ^26^, ^29^ (OR: 2.24, 95% CI: [1.46, 3.42], *p* < .0001) and (5) Intuity‐only studies^31^, ^32^, ^33^, ^34^, ^35^ (OR: 2.57, 95% CI: [1.49, 4.41], *p* = .001), demonstrating a higher pacemaker rate in the RDAVR group. Propensity‐matched studies tended toward favoring conventional AVR but no statistically significant difference was observed (OR: 1.50, 95% CI: [0.79, 2.85], *p* = .212), while pooled analysis of observational studies maintained a significantly higher pacemaker rate after RDAVR (OR: 2.83, 95% CI: [2.05, 3.90], *p* < .0001). Overall, there was homogeneity in these analysis (*I* ^2^ = 0%, *p* = .662).

### 3.5 ITU stay

**Figure 4:**
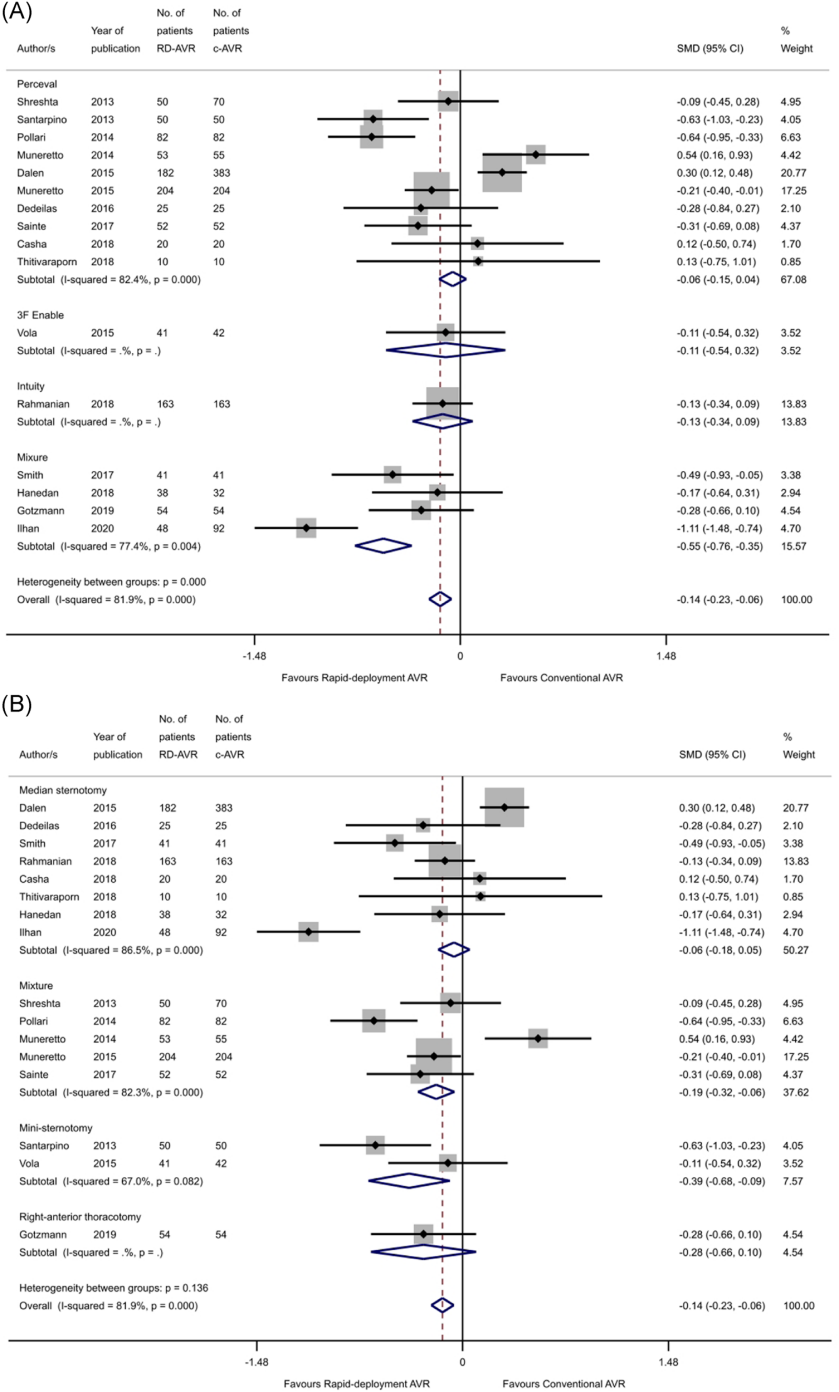
Forest plot demonstrating the ITU stay following RDAVR compared with cAVR. Subgroup analysis is shown based on (A) the RDAVR prosthesis used in each study, and (B) the surgical approach. cAVR, conventional aortic valve replacement; RDAVR, rapid deployment aortic valve replacement

Sixteen studies^7^, ^14^, ^21^, ^22^, ^23^, ^24^, ^25^, ^26^, ^28^, ^30^, ^34^, ^39^, ^40^, ^41^, ^43^, ^44^ included information about length of ITU stay. ITU stay was shorter for the RDAVR group (SMD: −0.14, 95% CI: [−0.23, −0.06], *p* < .001), although there was considerable heterogeneity (*I* ^2^ > 81.9%, *p* < .001) for this analysis (Figure 4).

This benefit persisted when analyzing mini‐sternotomy only studies (SMD: −0.39, 95% CI: [−0.68, −0.09], *p* = .01) and studies with a mixture of surgical approaches (SMD: −0.19, 95% CI: [−0.32, −0.06] *p* = .005). However, the between‐group difference was eliminated when analyzing sternotomy‐only studies (SMD: −0.06, 95% CI: [−0.18, 0.05], *p* = .284).

Subgroup analysis revealed no difference between cAVR and RDAVR when perceval‐only studies^7^, ^14^, ^21^, ^22^, ^23^, ^24^, ^25^, ^26^, ^28^, ^30^ were analyzed (SMD: −0.06, 95% CI: [−0.15, 0.04], *p* = .271), although studies analyzing a mixture of RDAVR types^39^, ^40^, ^41^, ^43^ did find a significantly shorter ITU stay in the RDAVR group (SMD: −0.55, 95% CI: [−0.76, −0.35], *p* < .001).

### 3.6 Echocardiographic follow‐up

#### 3.6.1 PVL

**Figure 5:**
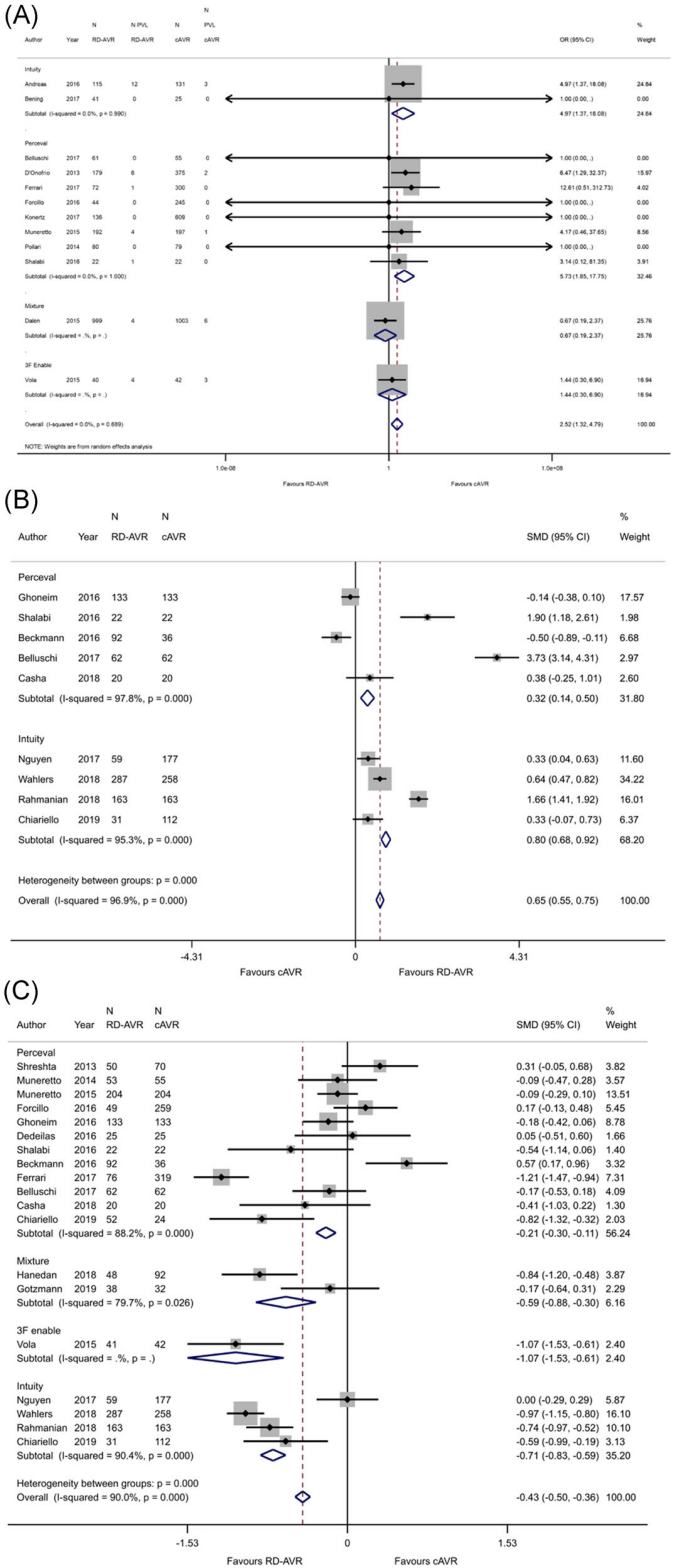
Echocardiographic outcomes, comparison of RDAVR with cAVR, including (A) paravalvular leak, (B) indexed effective orifice area, and (C) mean gradient across valve. cAVR, conventional aortic valve replacement; RDAVR, rapid deployment aortic valve replacement

Twelve studies^12^, ^13^, ^14^, ^15^, ^18^, ^21^, ^23^, ^27^, ^31^, ^32^, ^36^, ^44^ reported midterm data for echocardiographic evidence of at least moderate PVL. Pooled analysis demonstrated a higher incidence of PVL in the RDAVR group compared with cAVR (OR: 2.52, 95% CI: [1.32, 4.79], *p* = .005), with no evidence of heterogeneity (*I* ^2^ = 0%, *p* = .689) (Figure 5A). When stratified by sutureless valve type, the meta‐analysis of perceval‐only data (eight studies) found higher PVL in the RDAVR group (5.73, 95% CI: [1.85, 17.75], *p* = .002), while only two studies reported on Intuity‐only data, also with higher incidence of PVL in the RDAVR group (4.97, 95% CI: [1.37, 18.08], *p* = .015).

#### 3.6.2 iEOA

Nine studies^11^, ^12^, ^16^, ^27^, ^30^, ^33^, ^34^, ^35^, ^37^ included data on iEOA. When analyzed according to sutureless valve type (perceval and intuity), RDAVR was favored overall (OR: 0.65, 95% CI: [0.55, 0.75]). Heterogeneity was observed with both perceval and intuity studies (*I* ^2^ = 96.9%) (Figure 5B).

#### 3.6.3 Mean gradient across valve

Eighteen studies^7^, ^11^, ^12^, ^15^, ^16^, ^21^, ^22^, ^27^, ^28^, ^30^, ^32^, ^33^, ^34^, ^35^, ^37^, ^39^, ^40^, ^44^ reported data on mean gradient across the valve, of which 12 reported data on perceval valves, 2 on a mixture, and 4 on intuity. RDAVR was favored overall (OR: −0.43, 95% CI: [−0.50, −0.36]), and within subgroups. There was a significant amount of heterogeneity within subgroups and overall (*I* ^2^ = 90%) (Figure 5C).

#### 3.6.4 Peak gradient across valve

Similarly, peak gradient across the valve favored RDAVR over cAVR (OR: 0.84, 95% CI: [0.76, 0.92]), and across the 15 included studies,^7^, ^11^, ^12^, ^16^, ^21^, ^22^, ^30^, ^32^, ^33^, ^34^, ^35^, ^37^, ^39^, ^40^, ^44^ there was considerable heterogeneity (*I* ^2^ = 81.5%).

### 3.7 Metaregression

The influence study variations in patient/operative covariates (age, body mass index [BMI], left ventricular ejection fraction, Euroscore, and valve size) on the on four outcomes of interest (mortality, stroke, pacemaker insertion rate, and PVL) were analyzed. The listed covariates were found to have no impact on any of these four outcomes (*p* > .05) (Table 2).

**Table 2:** Results of meta‐regression demonstrating the influence of covariates on four outcomes of interest: mortality, stroke, pacemaker rate, paravalvular leak (PVL)

Covariate	Coef	Standard error	95% CI	*p* value
Influence of covariates on operative mortality				
Age	0.035	0.052	−0.052 to 0.122	.419
BMI	0.009	0.151	−0.306 to 0.323	.953
LVEF	0.0042	0.033	−0.065 to 0.074	.899
Euroscore	0.0057	0.044	−0.092 to 0.103	.898
Influence of covariates on stroke				
Age	−0.067	0.065	−0.204 to 0.069	.314
BMI	−0.117	0.250	−0.654 to 0.420	.649
LVEF	−0.039	0.018	−0.042 to 0.037	.892
Euroscore	−0.081	0.054	−0.208 to 0.045	.172
Influence of covariates on pacemaker rate				
Age	−0.004	0.037	−0.083 to 0.075	.916
BMI	0.020	0.126	−0.247 to 0.285	.878
LVEF	0.077	0.186	−0.724 to 0.880	.717
Euroscore	−0.002	0.064	−0.180 to 0.179	.997
Valve size	0.005	0.005	−0.009 to 0.020	.361
Influence of covariates on PVL rate				
Age	0.124	0.162	−0.575 to 0.822	.526
BMI	−0.635	0.567	−2.023 to 0.753	.306
LVEF	−0.0049	0.029	−0.097 to 0.088	.877
Euroscore	0.138	0.133	−0.435 to 0.711	.409
Valve size	−0.014	0.011	−0.048 to 0.020	.276

Abbreviation: BMI, body mass index. LVEF, left ventricular ejection fraction.

## 4 DISCUSSION

Rapid deployment valve technology (RDAVR—or sutureless valves) emerged in the last two decades based on the design of transcatheter prostheses. The technology these class of valves exhibit afford it two main recognized benefits: (i) ergonomic implantation and (ii) favorable valve hemodynamics. The valve design eliminates the need for sutures to be placed in the aortic annulus (typically only three guiding sutures required), which can typically take 15–20 min. Instead, the prosthesis is placed into position and expanded to take the shape of the left ventricular outflow tract/annulus where it sits.

The self‐expandable, stentless and sutureless Perceval S (Sorin Group Italia Srl) and the balloon‐expandable, stented intuity valve (Edwards Lifesciences) are the most frequently implanted RDAVR prostheses worldwide. In a recent study^45^ comparing the outcomes of intuity versus perceval (117 vs. 39), discharge echocardiography found iEOA to be higher in the intuity group, but peak or mean pressure gradients were comparable between groups, although no long‐term follow‐up results were reported. Operative mortality, pacemaker implantation, and operative times were comparable between the two groups.^45^

### 4.1 Advantages of RDAVR over cAVR

This study found an overall reduced AXT and CPB time afforded by the use of RDAVR. Average AXT for isolated RDAVR has been reported to be 42–46 min, and 56–57 min in operations with concomitant procedures.^46^, ^47^ Our findings correlate with the meta‐analysis by Sohn and colleagues^48^ who had similar findings, although subgroup analyses were not conducted. With subgrouping, the present analysis found that RDAVR caused further reduction in operative time in the context of 3 F enable (vs. other prostheses) and mini‐sternotomy (vs. other approaches) (displayed by more negative coefficients in the analysis). In other words, RDAVR perform best at reducing operative time compared with cAVR when performed in the minimally invasive setting.

Whether reduced operative time provides a measurable clinical benefit remains a matter of debate in the context of RDAVR versus cAVR. The theoretical benefits of reduced CPB and AXT times include a reduced risk of systemic inflammatory response, kidney injury, and coagulopathy, as ascertained from major cardiac procedures and trials comparing off‐pump with on‐pump coronary surgery.^49^ Indeed, reduced ITU stay was a significant finding in this study. Although further ITU‐specific data were not available for meta‐analysis, such as ventilator time and the incidence of complications (e.g., pneumonia and renal failure), the significantly reduced time in ITU acts as a surrogate for reduced complications in the RDAVR group. As well as patient benefits, reduced hospital stay in the context of AVR can contribute to recognizable cost‐benefits.^50^

### 4.2 Pacemaker incidence

In many studies (including those in this analysis), permanent pacemaker (PPM) implantation is used as a surrogate for the incidence of grade III atrioventricular block. Although it serves as a reliable and measurable outcome, it should be noted that institutions vary on the guidance for PPM insertion and their use can also be warranted for other cardiac disrhythmias, thus creating a potential source of heterogeneity.

Despite this, meta‐analysis of PPM implantation revealed no evidence of heterogeneity (*I* ^2^ = 0%, *p *= .662), thus suggesting, statistically, potentially consistent PPM protocols across the studies. From our findings, RDAVR unfortunately performs worse with regards the rate of PPM insertion in the early postoperative phase, reflecting compression of calcium against the bundle branch located at the base of the interleaflet triangle between the right and noncoronary cusps and the right fibrous trigone—leading to heart block. As RDAVR prostheses are designed to sit below the level of the aortic annulus, similar to the design of TAVI prostheses,^51^ this complication is more likely,^52^, ^53^ in contrast with conventional valves which are implanted in the supra‐ or intra‐annular position. The incidence is increased in the event of valve oversizing, which is often performed to offset the risk of para‐valvular leakage,^54^ especially in the hands of surgeons who in the early phases of RDAVR implementation at their respective units.^55^

### 4.3 Echocardiographic outcomes

PVL has a recognized incidence following RDAVR, also confirmed in this study—more than the risk post‐cAVR. Although data on patient functional status were not available for meta‐analysis, untreated severe PVL has been shown in some cases to result in left ventricle pressure and volume overload with leading to symptoms of heart failure, especially if the receiving chamber is noncompliant.^56^ In spite of this, the hemodynamic benefits of RDAVR have also been confirmed, namely in the form of better iEOA and transvalvular gradient compared with cAVR. This has particular benefit in patients at risk of patient‐prosthesis mismatch, especially in the context of small aortic roots.^12^ These findings emphasize the need for accurate patient selection in the context of AVR prosthesis choice, tailored toward annular profile, patient habitus, and functional status.

### 4.4 RDAVR compared with TAVI

According to National Audit Data, the number of surgical AVR has been increasing in steadily over the last decade. This is despite the recent expansion of risk‐categories encompassed by TAVI treatment, which continues to provide a significant alternative to all forms of surgical AVR, by avoiding sternal trauma altogether.

RDAVR has the potential to facilitate minimally invasive AVR through the ease of implantation and avoidance of annular stitching. When compared with TAVI, a previously reported propensity‐matched analysis (*n* = TAVI 538 vs. sutureless 385) demonstrated improved long‐term outcomes in RDAVR compared with TAVI, despite the increased need for blood transfusions in the short term.^57^ Furthermore, one randomized trial of TAVI with an early‐generation valve in 280 patients demonstrated that TAVI was not inferior to surgery with more than 5 years of follow‐up.^58^ Meta‐analysis found that sutureless valves result in improved perioperative survival compared with TAVI, albeit with only six studies analyzed, adding further weight to using sutureless valves as a viable option, especially for minimally invasive approaches.^59^

### 4.5 Strengths and limitations

This study benefits from strict inclusion criteria, a large pooled analysis and subgrouping according to clinically relevant variables. However, the findings should be taken with some caution. The statistical heterogeneity may reflect the differing pathologies between patient groups, including the size of aortic roots. Despite the increased risk of midterm PVL detected echocardiographically, studies lacked data on functional outcome in these patients. Finally, all included studies were comparative, however the main limitation in the present work is the lack of randomized studies (only one valid RCT was incorporated).

### 4.6 Conclusion

Despite the benefits of RDAVR in terms of reduced operative time and enhanced recovery, the risk of pacemaker insertion in the short‐term, and PVL in the long‐term, remains a significant cause for concern. There is a strong need for larger multicentre RCTs with long‐term follow‐up to provide conclusive evidence about the safety and efficacy of RDAVR.

## CONFLICT OF INTERESTS

George Asimakopoulos is a proctor for Perceval.

### AUTHOR CONTRIBUTIONS

All authors take responsibility for all aspects of the reliability and freedom from bias of the data presented and their discussed interpretation.

## APPENDIX A

### A.1

I. **Full search strategy**
  **EMBASE**
  1. exp sutureless technique/
  2. Sutureless.mp. [mp=title, abstract, heading word, drug trade name, original title, device manufacturer, drug manufacturer, device trade name, keyword, floating subheading word, candidate term word]
  3. Perceval.mp.
  4. intuity.mp.
  5. ((rdavr or rapid deployment) adj2 (aorta valve or aortic valve)).mp.
  6. su‐avr.mp.
  7. 1 or 2 or 3 or 4 or 5 or 6
  8. cavr.mp.
  9. conventional aortic valve replacement.mp.
  10. standard aortic valve replacement.mp.
  11. 8 or 9 or 10
  12. av replacement.mp. [mp=title, abstract, heading word, drug trade name, original title, device manufacturer, drug manufacturer, device trade name, keyword, floating subheading word, candidate term word]
  13. avr.mp. [mp=title, abstract, heading word, drug trade name, original title, device manufacturer, drug manufacturer, device trade name, keyword, floating subheading word, candidate term word]
  14. aortic valve prosthesis.mp. [mp=title, abstract, heading word, drug trade name, original title, device manufacturer, drug manufacturer, device trade name, keyword, floating subheading word, candidate term word]
  15. aortic prosthesis.mp. [mp=title, abstract, heading word, drug trade name, original title, device manufacturer, drug manufacturer, device trade name, keyword, floating subheading word, candidate term word]
  16. prosthetic aortic valve.mp. [mp=title, abstract, heading word, drug trade name, original title, device manufacturer, drug manufacturer, device trade name, keyword, floating subheading word, candidate term word]
  17. heart valve prosthesis implant*.mp. [mp=title, abstract, heading word, drug trade name, original title, device manufacturer, drug manufacturer, device trade name, keyword, floating subheading word, candidate term word]
  18. aortic stenosis repair.mp. [mp=title, abstract, heading word, drug trade name, original title, device manufacturer, drug manufacturer, device trade name, keyword, floating subheading word, candidate term word]
  19. ((surgery or surgeries or operation* or implant* or procedure* or repair* or replacement*) adj3 (aortic valve or aorta valve)).tw.
  20. aortic valve.tw.
  21. exp aortic valve prosthesis/
  22. exp aorta valve prosthesis/or exp aortic valve replacement/or exp aorta valve replacement/
  23. exp aortic stenosis/or exp bioprosthesis/
  24. 7 or 11
  25. 12 or 13 or 14 or 15 or 16 or 17 or 18 or 19 or 20 or 21 or 22 or 23
  26. 24 and 25
  27. limit 26 to human
  28. limit 27 to english language
  29. limit 28 to dd=20000101‐20200531

**MEDLINE**

1. exp Sutureless Surgical Procedures/
2. Sutureless.mp. [mp=title, abstract, original title, name of substance word, subject heading word, floating sub‐heading word, keyword heading word, organism supplementary concept word, protocol supplementary concept word, rare disease supplementary concept word, unique identifier, synonyms]
3. Perceval.mp.
4. intuity.mp.
5. ((rdavr or rapid deployment) adj2 (aorta valve or aortic valve)). mp.
6. su‐avr.mp.
7. 1 or 2 or 3 or 4 or 5 or 6
8. cavr.mp.
9. conventional aortic valve replacement.mp.
10. standard aortic valve replacement.mp.
11. 8 or 9 or 10
12. av replacement.mp. [mp=title, abstract, original title, name of substance word, subject heading word, floating sub‐heading word, keyword heading word, organism supplementary concept word, protocol supplementary concept word, rare disease supplementary concept word, unique identifier, synonyms]
13. avr.mp. [mp=title, abstract, original title, name of substance word, subject heading word, floating sub‐heading word, keyword heading word, organism supplementary concept word, protocol supplementary concept word, rare disease supplementary concept word, unique identifier, synonyms]
14. aortic valve prosthesis.mp. [mp=title, abstract, original title, name of substance word, subject heading word, floating sub‐heading word, keyword heading word, organism supplementary concept word, protocol supplementary concept word, rare disease supplementary concept word, unique identifier, synonyms]
15. aortic prosthesis.mp. [mp=title, abstract, original title, name of substance word, subject heading word, floating sub‐heading word, keyword heading word, organism supplementary concept word, protocol supplementary concept word, rare disease supplementary concept word, unique identifier, synonyms]
16. prosthetic aortic valve.mp. [mp=title, abstract, original title, name of substance word, subject heading word, floating sub‐heading word, keyword heading word, organism supplementary concept word, protocol supplementary concept word, rare disease supplementary concept word, unique identifier, synonyms]
17. heart valve prosthesis implant*.mp. [mp=title, abstract, original title, name of substance word, subject heading word, floating sub‐heading word, keyword heading word, organism supplementary concept word, protocol supplementary concept word, rare disease supplementary concept word, unique identifier, synonyms]
18. aortic stenosis repair.mp. [mp=title, abstract, original title, name of substance word, subject heading word, floating sub‐heading word, keyword heading word, organism supplementary concept word, protocol supplementary concept word, rare disease supplementary concept word, unique identifier, synonyms]
19. ((surgery or surgeries or operation* or implant* or procedure* or repair* or replacement*) adj3 (aortic valve or aorta valve)).tw.
20. aortic valve.tw.
21. exp Aortic Valve Stenosis/
22. Heart Valve Prosthesis Implantation/or exp Suture Techniques/
23. exp aortic stenosis/or exp bioprosthesis/
24. 7 or 11
25. 12 or 13 or 14 or 15 or 16 or 17 or 18 or 19 or 20 or 21 or 22 or 23
26. 24 and 25
27. limit 26 to human
28. limit 27 to english language
29. limit 28 to dt=20000101‐20200531

II. **Full inclusion and exclusion criteria**

Studies to be included will be those written in English and published in a peer‐reviewed journal between January 1, 2000 and May 31, 2020.

Inclusion criteria:

- Written in English. This can include studies where the initial data collection was run in a different language but the data extraction and analysis were written in English.
- Published between January 1, 2000 and May 31, 2020.
- Published in a peer‐reviewed journal.
- The study cohort consisted of patients 18 years of age or older at the time of the operation.
- The study cohort consisted of patients who have aortic stenosis or other aortic valve disease which requires replacement using either sutureless technique or conventional aortic valve replacement.
- Outcome measures of complications, mortality or morbidity were a primary aim.
- At least 10 patients in the study cohort were undergoing AV replacement using sutureless technique or conventional valve replacement.
- The study included two treatment arms, sutureless aortic valve replacement and conventional sutured aortic valve replacement, which were being compared.
  Exclusion criteria:
- Not written in English.
- Not published in a peer‐reviewed journal, as well as abstracts without full articles, editorials, case reports, case series and conference proceedings.
- The study cohort consisted of patients younger than 18 years of age at the time of operation.

- The study cohort consisted of any non‐aortic valve replacement patients, or if non‐AVR patients were included along with AVR patients and were not analyzed as a separate group.
- Study cohorts also included patients undergoing transcatheter or transapical surgeries without separate analysis for the sutureless or conventional aortic valve replacement procedures.
